# Cerebro-Spinal Lymphocytes in General Paralysis of the Insane

**Published:** 1909-03-06

**Authors:** 


### Neurology.

### CEREBRO-SPINAL LYMPHOCYTES IN GENERAL PARALYSIS OF THE INSANE.

The difficulty of diagnosing general paralysis of the insane in its earlier stages is well enough known. Any additional aid in its diagnosis is welcome. Such aid appears to be afforded by microscopical examination of the cerebro-spinal fluid obtained by lumbar puncture. The fluid is centrifugalised, and the deposit is made into films and stained in the ordinary way. If the slide abounds in lymphocytes the argument is in favour of general paralysis of the insane, or at least of a syphilitic affection of the central nervous system, as the work of Widal, Joffroy, Ravaut, Sicard, Babinski, Nageotte, Séglas, and others testifies.

The following twelve consecutive lumbar puncture results from one asylum indicate what we refer to. Seven patients afflicted with *dementia præcox* yielded a liquid which contained no more lymphocytes than does normal cerebro-spinal fluid. One case of chronic alcoholism with enfeeblement of the intellect, ideas of persecution and of grandeur, and epileptiform attacks, likewise presented no lymphocytosis.

The same absence of lymphocytosis in the cerebro-spinal fluid was found in a man, aged thirty-six, who had had syphilis six years before and his first epileptiform seizure a few days previously. In two general paralytics, on the other hand, the lymphocytosis was very marked. One of these patients had not at first been regarded as a case of general paralysis, but the subsequent course proved the correctness of the diagnosis. Another case of general paralysis in a stage of remission still gave a lymphocytosis, but less obviously than the first two. It was the second remission the patient had had, the only symptoms at the time being a state of enfeebled intellect, Robertson's sign, speech defects, and tremors of the hands; but he had been admitted in a state of agitated mania with ideas of grandeur.

A young man of 26 who had had syphilis five years before, and who had now suffered from very severe pains in the head for some time, began to present evidence of intellectual excitation with ambitious tendencies, and his cerebro-spinal fluid proved to be rich in lymphocytes. The previous year he had had a milder but similar attack, accompanied by transient paralysis. He now presented hardly any physical signs except a marked tremor of the hands. The diagnosis appeared difficult enough, but the lymphocytosis assisted materially in arriving at it.

Another patient, a man aged 44, who had had syphilis fifteen years previously and now had definite symptoms of general paralysis, also gave a marked cerebro-spinal lymphocytosis.

Thus, in eight cases of mental affections other than general paralysis, the cyto-diagnosis was negative in all eight; in five cases in which general paralysis was either certain or probable, the results were all five positive. There are certain to be exceptions, of course, and time will show what are the limitations of the test; but when it is remembered how closely the symptoms of dementia præcox, for example, may simulate those of general paralysis of the insane it is important to know that the cerebro-spinal fluid exhibits no excess of lymphocytes in the former as a rule, whereas it does so in an undoubted manner in the latter.

